# Cognitive Declines Precede and Predict Functional Declines in Aging and Alzheimer’s Disease

**DOI:** 10.1371/journal.pone.0073645

**Published:** 2013-09-02

**Authors:** Laura B. Zahodne, Jennifer J. Manly, Anna MacKay-Brandt, Yaakov Stern

**Affiliations:** Cognitive Neuroscience Division, Department of Neurology and Taub Institute for Research on Alzheimer’s Disease and the Aging Brain, Columbia University College of Physicians and Surgeons, New York, New York, United States of America; Chiba University Center for Forensic Mental Health, Japan

## Abstract

**Objective:**

To investigate the temporal ordering of cognitive and functional declines separately in older adults with or without Alzheimer’s disease (AD).

**Design and Setting:**

A community-based longitudinal study of aging and dementia in Northern Manhattan (Washington Heights/Hamilton Heights Inwood Columbia Aging Project) and a multicenter, clinic-based longitudinal study of prevalent AD at Columbia University Medical Center, Johns Hopkins School of Medicine, Massachusetts General Hospital, and the Hôpital de la Salpêtrière in Paris, France (the Predictors Study).

**Participants:**

3,443 initially non-demented older adults (612 with eventual incident dementia) and 517 patients with AD.

**Main Outcome Measures:**

Cognitive measures included the modified Mini-Mental State Exam and composite scores of memory and language derived from a standardized neuropsychological battery. Function was measured with the Blessed Dementia Rating Scale, completed by the participant (in the sample of non-demented older adults) or an informant (in the sample of prevalent AD patients). Data were analyzed with autoregressive cross-lagged panel analysis.

**Results:**

Cognitive scores more consistently predicted subsequent functional abilities than vice versa in non-demented older adults, participants with eventual incident dementia, and patients with prevalent AD.

**Conclusions:**

Cognitive declines appear to precede and cause functional declines prior to and following dementia diagnosis. Standardized neuropsychological tests are valid predictors of later functional changes in both non-demented and demented older adults.

## Introduction

Empirical investigation into the temporal ordering of Alzheimer’s disease (AD) signs has largely focused on cerebrospinal fluid and structural magnetic resonance imaging biomarkers [1,2]. Behavioral symptoms, including cognitive and functional decline, appear to follow these physiological changes in many patients [1].

Many investigators have assumed that cognitive changes precede functional changes prior to AD diagnosis [1]. Indeed, the concept of mild cognitive impairment (MCI) was proposed to describe early cognitive impairment prior to the manifestation of frank functional loss sufficient to warrant a diagnosis of dementia [3]. These MCI criteria required that functional abilities be “essentially normal.” Despite this criterion, numerous patients labeled with MCI exhibit some degree of functional impairment [4–6]. In recognition of these observations, the most recent recommendations of a National Institute on Aging and Alzheimer’s Association work group explicitly allow for functional problems in the diagnosis of MCI due to AD. These new criteria require only that patients “generally maintain their independence of function in daily life, with minimal aids or assistance,” thus blurring the temporal ordering of cognitive and functional decline in older adults at risk for dementia [7].

There have been few direct investigations of the temporal ordering of cognitive and functional declines prior to AD. Amieva and colleagues reported descriptively that declines in semantic memory and conceptual formation appeared to precede declines in activities of daily living in a sample of 350 AD converters [8]. In contrast, a recent community-based longitudinal study reported that baseline difficulties with certain informant-reported instrumental activities of daily living among cognitively intact individuals predicted MCI two years later [9]. Further, it has been suggested that a structured clinical interview focusing on functional changes is capable of diagnosing AD prior to the inflection point of cognitive decline [10]. Indeed, standardized neuropsychological tests have been criticized in their ability to predict future functional declines [11]. Thus, it remains possible that functional changes precede measurable cognitive changes, or that there is reciprocal causality between cognition and function prior to dementia onset.

The temporal ordering of cognitive and functional declines *following* AD diagnosis has largely been ignored. We recently demonstrated strong coupling of cognitive and functional declines both between and within patients diagnosed with probable AD [12]. However, whether there are leading and lagging relationships within those parallel trajectories remains unclear.

The purpose of this study was to investigate the temporal ordering of cognitive and functional declines separately in older adults with or without AD. In both samples, cognition was measured with neuropsychological tests, and function was measured using a self-report questionnaire completed by either the individual (in the sample of non-demented older adults) or an informant (in the sample of AD patients). We used autoregressive cross-lagged panel analysis to compare the predictive abilities of the cognitive variables for function versus functional variables for cognition. This approach allows for an exploration of reciprocal causality between cognition and function.

## Methods

Data from two longitudinal studies were used in the present analyses. A sample of initially non-demented older adults was drawn from the Washington Heights/Hamilton Heights Inwood Columbia Aging Project (WHICAP) [13,14]. A sample of prevalent Alzheimer’s disease (AD) cases was drawn from The Predictors Study, a multicenter study of AD progression [15].

### Ethics Statement

The local Institutional Review Board (IRB) at Columbia University approved the WHICAP study. Written informed consent was obtained from all WHICAP participants. Local IRB at all participating sites approved The Predictors Study. Written informed consent was obtained directly from patients at study entry. Patient assent was documented at each subsequent visit in accordance with IRB requirements.

### WHICAP

#### Participants and procedures

WHICAP is a prospective, community-based study of aging and dementia in a racially and ethnically diverse sample of Medicare-eligible residents of Northern Manhattan. Participants were identified from Medicare records and recruited in two waves: 1992 (N=1,486) and 1999 (N=1,957). Ongoing follow-up occurs at 18-24 month intervals and includes a battery of cognitive, functional, and health measures administered in the participant’s preferred language (English or Spanish). We have previously demonstrated acceptable measurement and structural invariance of our neuropsychological battery across English and Spanish speakers [16].

The current sample included 3,443 participants who did not meet criteria for dementia at their initial study visit. Dementia was determined by a consensus of a group of neurologists and neuropsychologists using *Diagnostic and Statistical Manual of Mental Disorders, Revised Third Edition* criteria [17]. To maximize the available data, only data from the first four occasions were analyzed for all participants, corresponding to an average follow-up period of 5.1 years (SD =2.4). The median number of visits was 3. Average time between assessments was 2.2 years (SD =1.0). Among the 612 participants who converted to dementia during the entire WHICAP study period (up to 22 years), 230 converted at the second study visit, 133 converted at the third study visit, 108 converted at the fourth study visit, and 140 converted at a future study visit (up to 22 years). Because only the first four visits were analyzed, data from the visit at which these 140 participants converted to dementia was not analyzed. Characteristics of the WHICAP sample are available in Table 1.

**Table 1:** Patient Characteristics at Study Enrollment.

	WHICAP total	WHICAP incident	Predictors
	(N=3,443)	(N=612)	(N=517)
Age	76.3 (6.4)	78.29 (6.8)	74.2 (8.5)
Education	9.8 (4.8)	7.5 (4.7)	13.7 (3.6)
Sex (% Female)	67.4	72.2	56.9
Race (% White)	27.1	13.6	93.2
Ethnicity (% Non-Hispanic)	60.3	55.4	95.2
Anti-dementia medications (% yes)	0.8	1.1	31.1^a^

Note. WHICAP=Washington/Hamilton Heights Inwood Columbia Aging Project, Predictors = The Predictors Study of Alzheimer’s disease.

^a^ Only participants recruited after 1997 reported taking anti-dementia medication at study entry.

#### Measures

Cognition was evaluated with tests of episodic memory and language. Episodic memory was chosen due to its central role in dementia progression. Language was chosen based on evidence that verbal fluency and verbal abstraction evidence the earliest declines prior to dementia diagnosis [8]. Tests were grouped into cognitive domains based on previous factor analysis of the WHICAP battery [16]. Raw scores at each occasion were standardized to T-score metric using the sample’s means and standard deviations at the initial occasion. Composite scores for each occasion were computed by averaging the three T-scores at that occasion within each domain.

The episodic memory domain included measures from the Selective Reminding Test (SRT) [18]. Participants are given six trials to learn a list of 12 words. Following each trial, participants are only reminded of the words they failed to recall. Total learning is quantified as the total number of words recalled after the six learning trials. Delayed recall and recognition are quantified as the number of words recalled or recognized after a 15-minute delay.

The language domain included measures of naming, verbal fluency, verbal abstraction, repetition, and comprehension. Naming ability was quantified as the number of spontaneously-identified objects on a 15-item version of the Boston Naming Test [19]. Verbal fluency was assessed for both letters and animals. Verbal abstraction was assessed with the Similarities subtest of the Wechsler Adult Intelligence Scale – Revised [20]. Repetition and comprehension were assessed with subtests of the Boston Diagnostic Aphasia Examination [21].

Functional abilities were assessed with items from the Blessed Dementia Rating Scale (BDRS) [22]. In WHICAP, the BDRS is a self-report instrument that asks about difficulties performing various activities of daily living for non-physical reasons. The sum of items specifically assessing instrumental (1,2,4,5) and basic (9–11) activities of daily living were used. Scores range from 0 to 13, with higher scores indicating greater functional difficulty. Scores on the cognitive and functional measures in the WHICAP sample are shown in Table 2.

**Table 2:** Scores at Each Visit.

	Memory	Language	mMMS	BDRS
WHICAP				
Visit 1	50.1 (8.4)	50.1 (7.5)	-	0.7 (1.1)
Visit 2	48.0 (10.4)	49.9 (7.9)	-	0.7 (1.6)
Visit 3	47.5 (10.5)	50.1 (7.7)	-	0.7 (1.7)
Visit 4	47.6 (10.5)	49.3 (8.1)	-	0.9 (1.8)
The Predictors Study				
Visit 1	-	-	37.6 (6.4)	1.9 (1.7)
Visit 2	-	-	35.4 (8.5)	2.4 (2.1)
Visit 3	-	-	33.0 (10.1)	3.1 (2.7
Visit 4	-	-	30.8 (10.9)	3.8 (3.1)
Visit 5	-	-	29.4 (12.0)	4.2 (3.2)
Visit 6	-	-	27.3 (12.9)	4.9 (3.5)
Visit 7	-	-	25.6 (14.2)	5.4 (3.7)
Visit 8	-	-	24.6 (13.8)	5.8 (3.6)
Visit 9	-	-	24.0 (14.2)	6.3 (3.8)
Visit 10	-	-	22.0 (15.1)	6.9 (3.9)
Visit 11	-	-	22.9 (14.9)	7.1 (3.9)
Visit 12	-	-	22.2 (14.0)	7.7 (3.9)

Note. WHICAP= WHICAP=Washington/Hamilton Heights Inwood Columbia Aging Project, mMMS = Modified Mini-Mental State Exam, BDRS = Blessed Dementia Rating Scale.

#### Covariates

In line with prior work with this dataset, models controlled for age, sex, race, ethnicity, education, recruitment year, and illness burden. Race and ethnicity were determined via self-report using the format of the 2000 US Census. To quantify illness burden at each occasion, one point was assigned for the presence of each of the following conditions: heart disease, hypertension, stroke, diabetes, pulmonary disease, thyroid disease, liver disease, renal insufficiency, peptic ulcer disease, peripheral vascular disease, cancer, Parkinson’s disease, Essential Tremor, Multiple Sclerosis, and arthritis. These points were summed to create an index of illness burden at each occasion.

### The Predictors Study

#### Participants and procedures

The current sample included 517 patients with probable AD recruited in two waves beginning in 1989 (N=252) or 1998 (N=265) from clinics at four sites: Columbia University Medical Center (N=208), Johns Hopkins School of Medicine (N=147), Massachusetts General Hospital (N=124), and the Hôpital de la Salpêtrière in Paris, France (N=38). Diagnoses of probable AD were made using NINCDS-ADRDA criteria [23] at consensus conferences. At enrollment, all patients were required to have mild dementia (i.e., score above 29 on the 57-point Modified Mini Mental State Exam, described below) and at least one family member/caregiver available. Exclusion criteria were non-AD dementia, parkinsonism, stroke, alcoholism, schizophrenia, schizoaffective disorder, and electroconvulsive treatments. Participants were assessed every 6-months. At least one follow-up assessment was available for 97% of the present sample. On average, patients were assessed on 10 occasions (SD=5.9). Only data from the first 12 occasions (six years) were included in the present study to maximize available data. Average attrition rate between visits was 8.5%, and 176 participants were assessed at the last visit. Characteristics of the Predictors sample are shown in Table 1.

#### Measures

Cognition was measured using the Modified Mini Mental State Exam (mMMS) [24]. In addition to items from the Mini Mental State Exam [25], the mMMS includes items assessing working memory, calculation, recall of the current and four previous presidents of the United States, confrontation naming, repetition, and visuoconstruction. The scale was translated and modified for assessments at the Paris site. Scores range from 0 to 57, with higher scores indicating better cognitive functioning.

Functional abilities were assessed with items from the BDRS [22]. In The Predictors Study, the BDRS is a semi-structured interview conducted with an informant that assess a patient’s difficulties performing various activities of daily living for non-physical reasons. As described above, the sum of items specifically assessing instrumental and basic activities of daily living were used. Scores range from 0 to 13, with higher scores indicating greater functional difficulty. Scores on the cognitive and functional measures in The Predictors Study sample are shown in Table 2.

#### Covariates

In line with prior work with this dataset, the Predictors model controlled for age, sex, education, and recruitment site. In a subset of 265 participants recruited in 1998, data were available to quantify illness burden at each occasion. Specifically, one point was assigned for the presence of each of the following conditions: hypertension, diabetes, myocardial infarction, congestive heart failure, valvular heart disease, angina, atrial fibrillation, other cardiac arrhythmia, hypercholesterolemia, hypertriglyceridemia, epilepsy, head trauma with loss of consciousness, pulmonary disease, thyroid disease, chronic liver disease, chronic renal disease, systemic malignancy, autoimmune disorder, and syphilis. These points were summed to create an index of illness burden at each occasion.

### Statistical analysis

Descriptive statistics were computed in SPSS version 19 (IBM Corp., Amonk, NY). Bivariate autoregressive cross-lagged panel analyses were conducted in Mplus version 7 (Muthén & Muthén, Los Angeles, CA) using maximum likelihood estimation. Missing data were managed with full information maximum likelihood (FIML), which uses all available data for parameter estimation.

**Figure 1:**
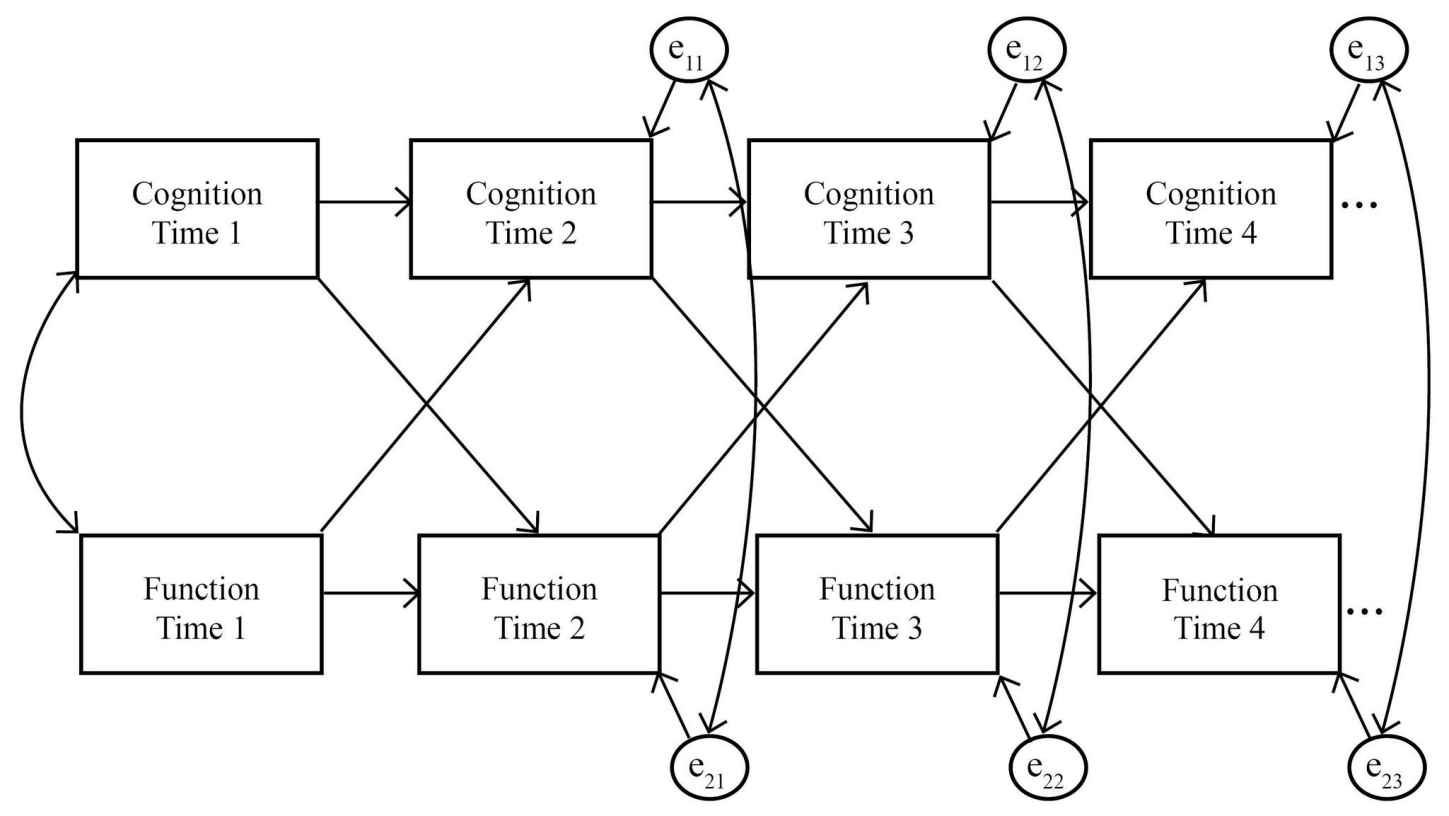
Schematic of the autoregressive cross-lagged model. Four time points were analyzed in the WHICAP models. Twelve time points were analyzed in the Predictors model. Refer to the text for the covariates included in each model (not shown).

In the cross-lagged models, each variable at each occasion was regressed onto the same variable at the previous occasion (autoregressive paths) as well as the other variable of interest at the previous occasion (cross-lagged paths). In addition, occasion-specific correlations between the two variables of interest were estimated. A schematic of the general model is shown in Figure 1. This framework allows for an exploration of reciprocal causality between cognitive and functional variables.

In the current study, three cross-lagged models were estimated. The first two examined relationships between function and one of the two cognitive abilities in WHICAP (i.e., memory or language) over an average of five years. In addition, these models were re-run on the subset of 612 individuals who converted to dementia during the study period in order to more specifically examine temporal ordering prior to confirmed dementia onset. The third model examined the relationship between function and global cognitive status in The Predictors Study over 5.5 years. In the subset of 265 participants recruited in 1998, a supplementary model was run in which a time-varying covariate reflecting illness burden was added to the model to confirm that results were not dramatically altered by the consideration of comorbid health conditions. Due to this smaller sample size used in this analysis, only 2.5 years of data were evaluated. Model fit was evaluated with the root mean square error of approximation (RMSEA<0.08), standardized root mean square residual (SRMR<0.05), and the comparative fit index (CFI>.95).

## Results

### WHICAP

Memory (RMSEA=0.05, SRMR=0.03, CFI=0.95) and language (RMSEA=0.05, SRMR=0.03, CFI=0.97) models fit well. After controlling for covariates, better functional ability was associated with higher concomitant memory (pooled *r*=-0.32; all *p*’s<.001) and language (pooled *r*=-0.33; all *p*’s<.001) scores at all four visits. Autoregressive and cross-lagged paths in the two models are shown in Table 3. Memory and language scores predicted functional ability at each subsequent visit. In contrast, functional ability only predicted subsequent memory or language scores at two out of three follow-up visits. Standardized regression coefficients were larger for the prediction of functional ability by cognitive domain at each visit.

**Table 3:** Results from the WHICAP models.

		Model 1: Memory	Model 2: Language
		Estimate (SE)	Estimate (SE)
Predictors of function			
Visit 2 function on			
	Visit 1 function	0.59** (0.02)	0.59** (0.02)
	Visit 1 cognition	-0.07** (0.02)	-0.12** (0.03)
Visit 3 function on			
	Visit 2 function	0.60** (0.02)	0.59** (0.02)
	Visit 2 cognition	-0.15** (0.02)	-0.13** (0.02)
Visit 4 function on			
	Visit 3 function	0.62** (0.02)	0.62** (0.02)
	Visit 3 cognition	-0.18** (0.03)	-0.19** (0.03)
Predictors of cognition			
Visit 2 cognition on			
	Visit 1 cognition	0.58** (0.01)	0.78** (0.01)
	Visit 1 function	-0.06** (0.02)	-0.06** (0.01)
Visit 3 cognition on			
	Visit 2 cognition	0.66** (0.02)	0.74** (0.01)
	Visit 2 function	-0.07* (0.02)	-0.04* (0.02)
Visit 4 cognition on			
	Visit 3 cognition	0.68** (0.02)	0.80** (0.02)
	Visit 3 function	-0.03 (0.03)	0.01 (0.03)

Note. SE = Standard Error. Models controlled for age, sex, race, ethnicity, education, and recruitment year. Presented estimates are in standardized metric.

* *p*<.05, **p<.001

In the restricted sample of incident dementia cases, memory (RMSEA=0.05, SRMR=0.05, CFI=0.96) and language (RMSEA=0.04, SRMR=0.05, CFI=0.97) models fit well. After controlling covariates, better functional ability was associated with higher memory scores at visits 2 through 4 (pooled *r*=-0.37; all *p*’s<.008). At the first visit, the association between function and memory was not significant (*r*=.10, *p*=.062). Better functional ability was also associated with higher language scores at the all four visits (pooled *r*=-0.35; all *p*’s <.05). With regard to cross-lagged paths, memory and language scores predicted subsequent functional ability at follow-up visits 3 (memory: beta=-0.10, *p*=.03, language: beta=-0.13, *p*=.005) and 4 (memory: beta=-0.20, *p*<.001, language: beta=-0.18, *p*=.002). In contrast, functional ability did not predict subsequent memory or language scores at any of the follow-up visits (all *p*’s >.07).

### The Predictors Study

The model fit well (RMSEA=0.04, SRMR=0.03, CFI=0.98). At all twelve occasions, better functional ability was associated with higher global cognitive status (pooled *r*=-0.64; all *p*’s<.001). With regard to the autoregressive paths, previous functional ability predicted subsequent functional ability at each visit (pooled beta=0.78, pooled SE= 0.03, all *p*’s<.001). Similarly, previous global cognitive status predicted subsequent global cognitive status at each visit (pooled beta=0.91, pooled SE= 0.02, all *p*’s<.001). Cross-lagged paths are shown in Table 4. As shown, global cognitive status predicted functional ability at each subsequent visit. In contrast, functional ability only predicted global cognitive status at three out of the 11 follow-up visits. Standardized regression coefficients were larger for the prediction of functional ability by cognition at each visit.

**Table 4:** Results from The Predictors Study model.

	Predictor of function	Predictor of global cognitive status
	Estimate (SE)	Estimate (SE)
Visit2 on Visit1	-0.15** (0.03)	-0.14** (0.03)
Visit3 on Visit2	-0.17** (0.03)	-0.05 (0.03)
Visit4 on Visit3	-0.16** (0.03)	-0.06* (0.03)
Visit5 on Visit4	-0.14** (0.03)	-0.07* (0.03)
Visit6 on Visit5	-0.20** (0.03)	0.02 (0.03)
Visit7 on Visit6	-0.07* (0.03)	-0.05 (0.03)
Visit8 on Visit7	-0.15** (0.03)	-0.00 (0.03)
Visit9 on Visit8	-0.18** (0.04)	0.01 (0.03)
Visit10 on Visit9	-0.13* (0.04)	0.03 (0.03)
Visit11 on Visit10	-0.15* (0.05)	0.00 (0.03)
Visit12 on Visit11	-0.25** (0.05)	0.03 (0.05)

Note. SE = Standard Error. Models controlled for age, sex, education, and recruitment site. Presented estimates are in standardized metric.

* *p*<.05, **p<.001

A supplementary model was run in the subset of 265 participants recruited in 1998 adding a time-varying covariate reflecting illness burden. This model fit well (RMSEA=0.07, SRMR=0.05, CFI=0.97). Better functional ability was associated with higher global cognitive status at the first five occasions (pooled *r*=-0.47; all *p*’s<.013). With regard to the autoregressive paths, previous functional ability predicted subsequent functional ability at each visit (pooled beta=0.75, pooled SE= 0.05, all *p*’s<.001). Similarly, previous global cognitive status predicted subsequent global cognitive status at each visit (pooled beta=0.88, pooled SE= 0.04, all *p*’s<.001). With regard to cross-lagged paths, global cognitive status predicted functional ability at each subsequent visit (pooled beta=-0.16, pooled SE= 0.06, all *p*’s<.03). In contrast, functional ability only predicted global cognitive status at the second visit (beta=-0.13, SE= 0.06, *p*=.02). Standardized regression coefficients were larger for the prediction of functional ability by cognition at each visit.

## Discussion

The main finding was that cognitive score more consistently predicted subsequent functional ability than vice versa in both non-demented older adults and patients with Alzheimer’s disease (AD). Among non-demented older adults, composites scores of memory or language reliably predicted self-reported functional ability at every subsequent visit, whereas self-reported functional ability only predicted subsequent cognitive scores two-thirds of the time. In addition, standardized regression coefficients were larger for the prediction of functional ability by cognitive domain. Restricting the sample to incident dementia cases, cognitive score predicted subsequent functional ability at two of the three follow-up visits, while functional scores did not predict subsequent cognitive scores at any visit. Among patients with prevalent AD, cognitive score predicted informant-rated functional ability at each subsequent visit, whereas functional ability only predicted subsequent cognition at three out of 11 follow-up visits. Further, the magnitude of association between cognitive score and subsequent functional ability was larger than that between functional score and subsequent cognition for every visit. These findings were independent of chronic illness burden and are most consistent with the view that cognitive declines precede functional declines.

These results validate the use of neuropsychological tests for functional prognosis among older adults with and without AD. It is notable that a previous study reporting poor prognostic ability of neuropsychological tests did not examine cognitive abilities assessed in the present study: word list learning, basic language ability, and verbal abstraction [11]. Together, these results support the importance of assessing memory and language with tests that require greater executive demands to detect the earliest deficits of AD [26].

Results provide minimal evidence for reciprocal causality between cognition and function and clarify our earlier demonstration of tight coupling of cognitive and functional declines over the course of AD by showing that decrements in the ability to perform tasks of daily living temporally follow and are predicted by decrements in cognition [12]. Thus, it appears likely that functional impairment in AD is a direct result of cognitive impairment.

Importantly, our finding that cognitive declines preceded functional declines in the sample of initially non-demented older adults was replicated, and even more pronounced, in the subset of participants who went on to develop dementia. The interaction between cognitive and functional decline appears to begin prior to formal dementia diagnosis. It has been suggested that the Clinical Dementia Rating (CDR), a comprehensive clinical interview focusing on real-world functioning, is sufficient to diagnose dementia prior to the inflection point of cognitive decline [10]. However, it should be noted that the CDR is much more than a functional scale. Not only does it assess activities of daily living, it includes a more wide-ranging clinical interview with both patient and knowledgable informant. It also involves direct interrogation of cognitive abilities (e.g., recall of a name and address). Such a comprehensive clinical interview supplemented by overt cognitive testing represents the gold standard in clinical diagnosis and prognosis. Our results show that in the absence of such thorough clinical assessment, standardized neuropsychological testing is also capable of predicting later changes in functioning.

There were minimal differences in the observed temporal associations in the two populations studied (a community-based study of initially non-demented older adults and a clinic-based study of older adults with prevalent AD). In the initially non-demented sample, associations between cognition and subsequent function were somewhat stronger for later time points, perhaps because these variables became more coupled as cognitive and functional abilities declined. Indeed, the magnitudes of association between the cognitive variables and subsequent functioning at these later time points were similar to those seen between global cognitive status and subsequent functioning in the prevalent AD sample.

Amieva and colleagues (2008) have descriptively shown that declines in cognitive performance appear to precede declines in activities of daily living prior to AD diagnosis [8]. The current findings extend this observation by showing that cognitive scores significantly and independently *predicted* functional scores at subsequent visits. While the magnitudes of the associations between cognition and subsequent function were similar at later visits, language scores at the initial study visit appeared to be a stronger predictor of functioning at the second visit than memory scores. These results may be consistent with those of Amieva et al. (2008), who reported that verbal fluency and verbal abstraction, both of which were included in our language composite, were the first tests to evidence declines prior to AD conversion in their French sample. Alternatively, function could have been more sensitive to declines in language than memory if both domains declined similarly in the present study.

A main strength of this study is its large numbers of older adults both prior to and following dementia onset. Another strength is the use of panel analysis, which allowed for a direct examination of the temporal ordering of cognitive and functional changes. While cognitive scores were limited to a single measure of global cognitive status in the AD sample, we were able to replicate our results with composite scores of memory and language tests in the sample of initially non-demented older adults. A limitation of analyses on the WHICAP sample was the lack of an informant-based measure of function, as the presence of a reliable informant was not an inclusion criterion for this community-based study. It should be noted that the same pattern of results (i.e., cognitive declines precede and predict functional declines) was found in the Predictors sample, in which function was measured via informant report.

In conclusion, the present study supports the view that cognitive declines precede and cause functional declines in late life. In addition, standardized neuropsychological tests represent a useful marker of functional prognosis during all stages of dementia. While the current study cannot address the temporal ordering of biomarkers as described by Jack et al. (2010, 2013) [1,2], our findings support the hypothesis that cognitive changes precede functional changes prior to AD diagnosis as depicted in that model.
